# Fibroblast growth factor-6 enhances *CDK2* and *MATK* expression in microvesicles derived from human stem cells extracted from exfoliated deciduous teeth

**DOI:** 10.12688/f1000research.14900.6

**Published:** 2020-06-24

**Authors:** Ahmed Othman, Rabab Mubarak, Dina Sabry

**Affiliations:** 1 Department of Oral Biology, Faculty of Oral and Dental Medicine, Cairo University, Cairo, Egypt; 2 Department of Medical Biochemistry and Molecular Biology, Faculty of Medicine, Cairo University, Cairo, Egypt

**Keywords:** Stem cells from human exfoliated deciduous teeth, fibroblast growth factor 6, microvesicles, CDK2, MAKT

## Abstract

**Background:** Stem cells from human exfoliated deciduous teeth (SHEDs) are considered one of the most convenient sources of adult stem cells. This study aimed to examine the effect of fibroblast growth factor 6 (FGF-6) on SHEDs and evaluate *CDK2* and *MATK* gene expression in SHED-derived microvesicles (MVs). SHEDs were cultured from deciduous teeth pulp.

**Methods:** SHEDs were divided into two groups: the control group and test groups, with and without FGF-6 supplementation, respectively. After the third passage, SHED proliferation was assessed by MTT assay. MVs were purified and *CDK2* and *MATK* gene expression was assessed by real-time polymerase chain reaction. SHEDs were identified by their positivity for CD90 and CD73, and negativity for CD45 and CD34.

**Results:** SHEDs proliferation in the test group was significantly higher than in the control group (P<0.001). mRNA from SHED-derived MVs from the test group exhibited a markedly elevated expression of *CDK2* and *MATK*, (P<0.002 and P<0.005, respectively) in comparison with those of the control group. FGF-6 enhanced the proliferation of SHEDs. Proliferation enhancement is favorable for the production of a large number of stem cells, which will then be beneficial for cell-based therapies.

**Conclusions:** *CDK2* and *MATK* genes in SHED-derived MVs can be used as molecular biomarkers for SHED proliferation.

## Introduction

Stem cells from human exfoliated deciduous teeth (SHEDs) are a type of adult stem cell acquired from the dental pulp of human exfoliated deciduous teeth. SHEDs stand out from other types of adult stem cells since they possess a remarkable growth and proliferation rate, providing an adequate stem cell source for any prospective clinical or laboratory use. The natural exfoliation of deciduous teeth provides a good chance to procure and isolate SHEDs without effort or complications, and with little or no trauma ^1– 3^.

Fibroblast growth factors (FGFs) are a family of secreted cytokine proteins that have a role in the regulation and direction of numerous cellular processes, including proliferation, differentiation, migration or metabolism ^4^. FGF-6 is part of the FGF-4 subfamily of canonical FGFs. Like the other members of FGF-4 subfamily, FGF-6 is a secreted protein with a cleavable N-terminal signal peptide that binds and activates FGF receptors as an extracellular mediator ^4^. Despite the fact that FGF-6 expression is almost completely limited to myogenesis ^5^, it was found to exert a proliferating effect on human osteoblasts under specific conditions ^6^.

Recently, microvesicles (MVs) have been identified as an approach deployed by stem cells as a mean of mediating intercellular interactions ^7^. These phospholipid membrane-bound MVs partake in intercellular interactions, such as proliferation, differentiation and gene expression alteration, via their content of mRNA, miRNA and transfer proteins ^7, 8^.

 Human liver stem cell-derived MVs were found to have a role in hepatic regeneration, since they transfer proteins and mRNAs associated with the control of transcription, translation, proliferation, and apoptosis to hepatocytes ^9^. Cyclin-dependent kinase 2 ( *CDK2*) and megakaryocyte-associated tyrosine kinase ( *MATK*) genes are associated with cellular proliferation as they were found in the mRNA of purified MVs at the time of microarray analysis and reverse transcription-quantitative PCR (RT-qPCR) ^9^.

CDK2 is the catalytic subunit of the cyclin-dependent protein kinase complex, which controls advancement through the cell cycle via its involvement in the G _1_ to S phase transition ^10, 11^. MATK has been identified by Avraham *et al.* as an intracellular tyrosine kinase ** that participates in the proliferation and survival of megakaryocyte progenitors ^12^. Furthermore, Findings by Herrera *et al.* demonstrated that *MATK* conveyed by MVs was one of the genes responsible of liver stem cell proliferation ^9^.

The current study was performed to use SHEDs derived microvesicles as biomarker for cellular proliferation after FGF-6 supplementation by assessing the *CDK2* and *MATK* gene expression in microvesicles’ mRNA.

## Methods

### Sample collection

A total of 28 deciduous teeth indicated for extraction were collected from 25 patients at the Pediatric Dentistry Department in Faculty of Dental Medicine, Cairo University. Patient age ranged from 7 to 12 years. Collection was done at the pediatric clinic over 3 days, we looked for deciduous teeth indicated for extraction due to their natural shedding time in order to make room for their permanent successors, so no ethical concerns would arise. Deciduous tooth collection was conducted after obtainment of the guardians’ written informed consent at Pediatric Dentistry Department in the Faculty of Dental Medicine Cairo University, with the approval of the Ethics Committee of the Faculty of Oral and Dental Medicine, Cairo University. Subjects were identified by their treating physician, following which we contacted the guardians of the subjects for consent to use the extracted teeth. Stem cell propagation (at the Medical Biochemistry Department in the Faculty of Medicine Cairo University) was performed in accordance with recommendations and with the approval of the Ethics Committee of the Faculty of Oral and Dental Medicine, Cairo University.

Deciduous tooth surfaces were washed several times with Dulbecco’s PBS (Biowest, USA). Dental pulp was extracted delicately from teeth using a sterile endodontic barbed broach and placed in falcon tube containing PBS (Biowest, USA).

### SHED culture and characterization

SHEDs culture and characterization were done after taking established procedures into account ^13^. A total of 3 mg collagenase type II (Sigma Aldrich, USA) was dissolved in PBS to digest the extracted dental pulp tissues for 1 h at 37°C in a 5% CO _2_ incubator and shaken well at 10 min intervals until the tissues were fully digested. The samples were strained using a cell strainer (40 µm nylon PP) (Bio Basic, Inc., Canada) to remove tissue debris and then centrifuged for 10 min at 3000 rpm at 5°C to obtain pellets of isolated cells. The supernatant fluid was discarded and cell suspension was obtained by pipetting cells in RPMI 1640 culture medium (Biowest, USA). Next, the isolated cell pellets were seeded in 75 cm ^3^ tissue culture flasks for cell culture propagation. Culture medium (RPMI 1640) (was supplemented with 1% Pen/Strep solution (Lonza, USA) and 10% fetal bovine serum (FBS) (Lonza, USA) were supplemented to the culture media to achieve cell propagation at 37°C in humidified CO _2 _incubator for 7–10 days, with medium changes every 3 days.

Cells were identified as being mesenchymal stem cells (MSCs) by their morphology and adherence to the plastic flask. In addition, quantification of several expressed MSCs markers was conducted using flow cytometry analysis. Adherent cells were trypsinized and subjected to centrifugation to form cell pellet. Next, 1×10 ^5^ cells were incubated with 10 μl monoclonal CD90 PE (catalog number FAB2067A; R&D Systems), CD73PE (catalogue number FAB5795P; R&D systems) CD34 PE (catalogue number FAB72271P; R&D Systems) and CD45 PE (catalog number DAB1430P; R&D Systems) antibodies, at 4°C in the dark. Same species isotypes served as a negative control, Mouse IgG1 PE conjugated antibody (catalog number IC002P; R&D Systems). After a 20 min incubation, 2 ml PBS containing 2% FBS was added to a tube of monoclonal treated cells. The mixtures were then centrifuged for 5 min at 2500 rpm, followed by discarding the supernatant and re-suspending cells in 500 μl PBS containing 2% FBS. Cell analysis was performed using a CYTOMICS FC 500 Flow Cytometer and analyzed using CXP Software version 2.2.

### SHEDs proliferation process and passaging

Passaging of SHEDs was done according to established protocols ^14^, with modifications for this experiment. Sub-culturing and passaging was done when adherent cells primary culture (passage zero) have reached 80% confluence. 10 ^3^–10 ^5^ cells were seeded into 24-well plates prior to grouping and subsequent passaging till reached third passages. Seeded cells were divided into two groups: control group (SHEDs untreated with FGF-6) and test group (SHEDs treated with FGF6). FGF-6 was added at concentration 20 ng/ml for test group.

### Cell viability

MTT reagent, supplied ready for use after the third passage of the SHEDs, was obtained from Tacs Trevigen (Gaithersburg, USA). For the cell viability assay, the two cell groups were seeded in three 96-well tissue culture plates each, at 10 ^3^ cells/ml per well. The MTT reagent was added and the plate was incubated in the dark for 2–4 h. Detergent reagent (catalog number # 4890-25-02, TACS) was added to each well to solubilize formazan dye prior to absorbance measurement. The absorbance in each well was measured at a range from 490 to 630 nm using an enzyme-linked immunosorbent assay plate reader (Stat Fax 2200, Awareness Technologies, Florida, USA) ^15^.

### MV isolation

MVs were obtained from supernatants of third-passage MSCs (5×10 ^6^ cells/ml) cultured in RPMI-1640 deprived of FBS and supplemented with 0.5% of bovine serum albumin (BSA) (Sigma Aldrich, USA). After centrifugation at 2000 *g* for 20 min to remove debris, cell-free supernatants were centrifuged at 100,000 *g* for 1 h at 4°C, washed in serum-free medium 199 containing 25 mM HEPES (Sigma) and submitted to a second ultracentrifugation under the same conditions ^16^. MVs were then prepared for electron microscopy characterization. Briefly MVs were diluted in 145 µL PBS containing 0.2% paraformaldehyde (w/v). 10 µl was administered to a formvar-carbon-coated 300 mesh grid (Electron Microscopy Sciences, Hatfield, USA) for 7 min, followed by staining with 1.75% uranyl acetate (w/v). Samples were left to dry at room temperature for 2 h and imaged by transmission electron microscopy (TEM) (CM-10, Philips, Eindhoven, The Netherlands) at 100 kV afterwards ^17^.

### Gene expression profile

Total RNA was isolated from MVs using an RNA purification kit (Gene JET, Kit, #K0731, Thermo Fisher Scientific, Inc.). RNA quantification using RT-qPCR was achieved using a one-step reaction (SensiFAST™ SYBR® Hi-ROX One-Step Kit, catalog no. PI-50217 V; Bioline, UK). Sequence-specific primers (Bio Basic, USA) for the studied target genes ( *CDK2* and *MATK*) and reference housekeeping gene (β-actin) were used. The prepared reaction mix samples were applied in real time PCR (StepOne Applied Biosystem, Foster city, USA). The cDNA was subsequently amplified using a SYBRGreen I PCR Master kit (Fermentas) in a 48-well plate as follows: 10 min at 95°C for enzyme activation, followed by 40 cycles of 15 s at 95°C, 20 s at 60°C and 30 s at 72°C for the amplification step. Changes in the expression of each target were normalized relative to the mean Cq values of β-actin as housekeeping gene by the 2 ^−∆∆Cq^ method. We used 1 µM of both primers specific for each target gene. Primers sequences were as follows: *CDK2* sense, 5'-AATCCGCCTGGACACTGAGA-3' and antisense, 5'-CCAGCAGCTTGACAATATTAGGA-3' (Genbank accession number XM011537732.1); *MATK* sense, 5'-CCGCGACGTCATCCACTAC-3' and antisense, 5'-TTGTAATGCTCCACCATGTCCAT-3' (Genbank accession number AH006874.3); and *β-actin* sense, 5′-GCCGGGACCTGACTGACTAC-3′ and antisense, 5′- TTCTCCTTAATGTCACGCACGAT-3′ (Genbank accession number NM001101.3).

### Statistical analysis

Data were coded and entered using SPSS version 23. Data are presented as the median and interquartile range for quantitative data Comparisons between quantitative variables were done using the non-parametric Mann-Whitney test. Correlations between quantitative variables were done using Spearman’s correlation coefficient. P-values less than 0.05 were considered as statistically significant.

## Results

### SHED characterization

**Figure 1.**
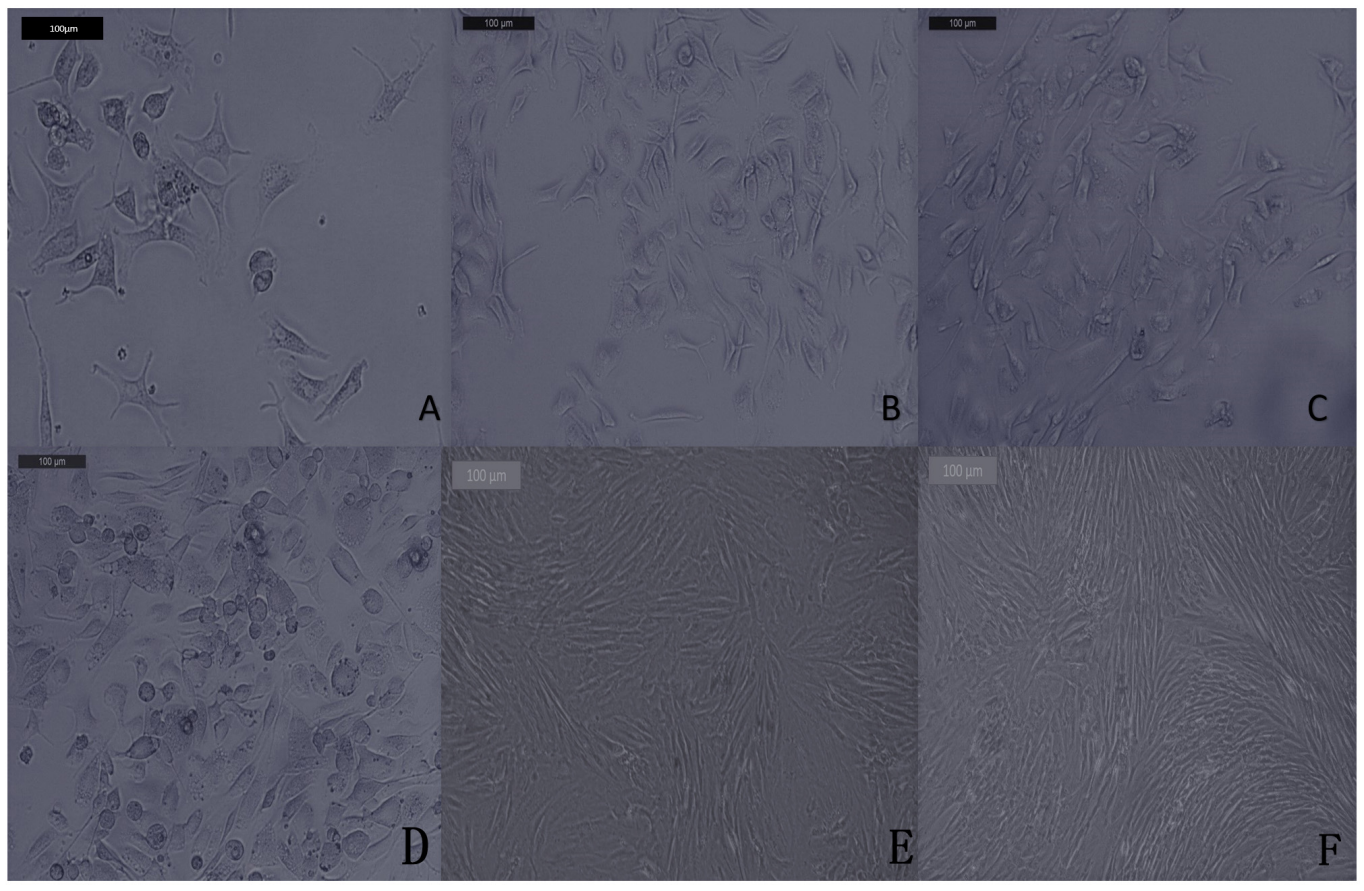
Isolation, morphological observation of stem cells from human exfoliated deciduous teeth through phase contrast microscopy. ( **A**) Passage one shows stem cells with spindle-like morphology as grow from human exfoliated deciduous teeth pulp in few number. ( **B** and **C**) Passages two ( **B**) and three ( **C**) show an increase in number of stem cells with spindle-like morphology. Isolation, morphological observation of stem cells from human exfoliated deciduous teeth in the test group through phase contrast microscopy. ( **D**) Passage one shows a marked increase in number and confluency of stem cells with spindle-like morphology in comparison with control group in passage one. ( **E** and **F**) Passages two ( **E**) and three ( **F**) show a pronounced, confluent and expanded SHED with fibroblast like morphology in relation to control groups of second and third passages.

**Figure 2.**
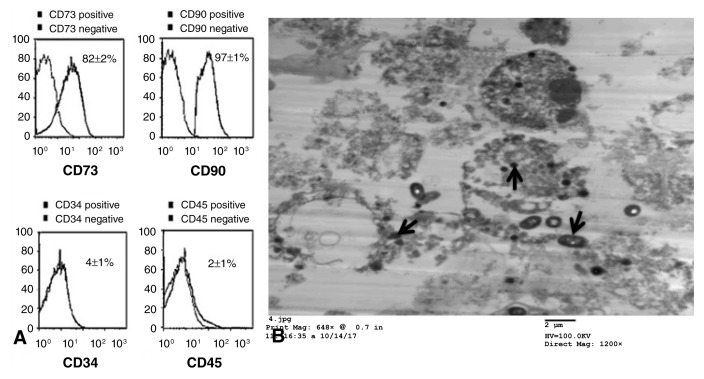
Flow cytometry and transmission electron microscopy. ( **A**) Flow cytometry analysis of CD90, CD73, CD34 and CD45 for stem cell characterization. ( **B**) Electron microscopy ultrastructure of released microvesicles (black arrow) from the mesenchymal stem cells of dental pulp.

Cultured SHEDs exhibited fusiform fibroblast like appearance for both groups. During culture and passaging, SHEDs in the test group proliferated more than SHEDs in the control group ( Figure 1). Flow cytometric analysis for SHEDs was negative for CD34 and CD45 and positive for CD90 and CD73 ( Figure 2A).

### Cell viability

The viability of the cells in the test group (n=17) was significantly higher (P<0.001) in comparison with that of the control group (n=17) ( Table 1).

**Table 1.** Cell proliferation assay for both studied groups. Data presented as median (IQR).

Variable	SHEDs	SHEDs supplemented with FGF-6	P-value
Absorbance (450 nm)	0.90 (0.77-1.36)	2.55 (1.63-2.98)	<0.001

SHEDs, stem cells from human exfoliated deciduous teeth; FGF-6, fibroblast growth factor-6.

### TEM

TEM detected MVs purified from SHED after ultracentrifugation ( Figure 2B). MVs were characterized by their size (500 nm), as detected by TEM.

### RT-qPCR

**Figure 3.**
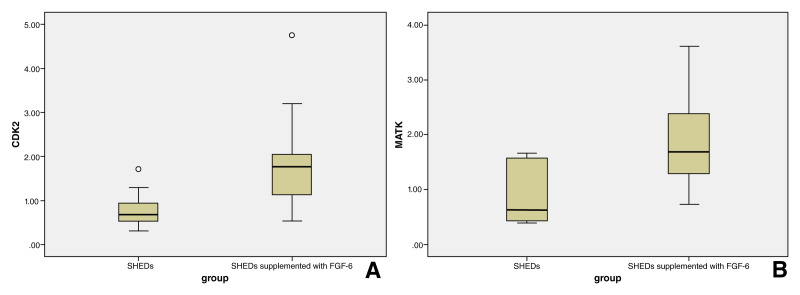
Box-and-whiskers plot showing number distribution for *CDK2* and *MATK* expression in both test and control groups. ( **A** and **B**) Expression of each gene is higher in the FGF-6-supplemented group than in the control group, since higher interquartile range (IQR) and median values are observed.

**Figure 4.**
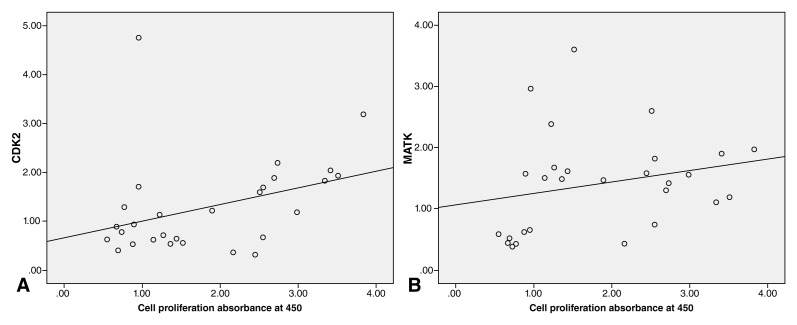
Assessment of cell proliferation. ( **A**) Expression of *CDK2* is positively correlated with cell proliferation in the test group (P=0.010) (r=0.480). ( **B**) Expression of *MATK* is positively correlated with cell proliferation in the test group (P=0.031) (r=0.409).

Purified MVs demonstrated a significant positive expression intensity of *CDK2* (P=0.002) (n=17), and *MATK* (P=0.005) (n=17) in the test group in relation with the control group. A box plot ( Figure 3) shows that expression of *CDK2* and *MATK* is higher in the test group than the control group, as they display a higher interquartile range (IQR) and medium. Expression of *CDK2* is positively correlated with cell proliferation in the test group (P=0.010) (r=0.480). Expression of *MATK* is positively correlated with cell proliferation in the test group (P=0.031) (r=0.409) ( Figure 4).

## Discussion

We performed this experiment to deal with difficulties sourcing stem cells and a lack of sufficient stem cells for reliable tissue formation. The study focused on stem cells isolated from human deciduous teeth (SHEDs) and tissue-inducing substances, which in this case is FGF-6. The reason we chose SHEDs for the isolated stem cells is that they present an opportune source of adult stem cells; the deciduous teeth are naturally exfoliating so there are no ethical problems surrounding their use, and the isolation of cells is simple, painless, convenient and time-efficient ^18^.

There are several criteria for SHED identification; we identified stem cells by their morphology under the inverted microscope, as they appeared as fibroblast-like cells. Another feature exhibited was that they have a plastic adherence feature under our normal culture conditions ^1^. SHEDs were also identified as ectomesenchymal stem cells through the quantification of several expressed mesenchymal stem cell markers using flow cytometry; they were shown to be positive for CD90 and CD73, and negative for CD45 and CD34 ^19^.

In this case, the stem cells are quiescent ^20^, unlike progenitor cells, meaning growth factor treatment is required to produce a large amount of cells. In our experiment, FGF-6 was chosen as it has, to our knowledge, never used on SHEDs before, and would avoid the conflicting reports of the effects of bFGF on SHEDs ^21, 22^.

SHEDs group with added FGF-6 demonstrated increased cells vitality and number in comparison with the control group. It was evidenced by the MTT assay results and increased expression of both CDK2 and MATK genes present in RNA of microvesicles we purified from SHEDs. We considered microvesicles as a good indicator or a biomarker for cellular proliferation of stem cells in general and SHEDs in particular. Other studies highlighted the importance of microvesicles ’cargo: They can be used as a biomarkers of tumor cells proliferation and progression, cardiometabolic disorders, immunologic diseases, and also cell-derived MVs are found to be able to change phenotypes of different cells to become stem cells via epigenetic reprograming or infectious particle transfer ^23– 27^. *CDK2* gene was used to obverse SHEDs proliferation ** since it has been used to monitor proliferation in many types of stem cells, such as neural progenitor stem cells ^28^ and liver stem cells ^9^. *CDK2* also encodes a serine/threonine protein kinase family member, with receptors in this family having a role in the regulation of cell proliferation, programmed cell death, cell differentiation, and embryonic development ^29^.

 Megakaryocyte-associated tyrosine kinase is the enzyme which is encoded by *MATK* in humans. This enzyme possesses a similar amino acid sequence to tyrosine-protein kinase CSK. It was chosen for our experiment as it is not frequently used for the assessment of SHED proliferation, to evaluate whether this ambiguous gene can be studied in further research to assess the proliferation rate of SHEDs and other types of stem cells it is known to be capable of phosphorylating and inactivating Src family kinases, and may inhibit T-cell proliferation ^12^.

## Conclusion

The present study showed an increased expression of CDK2 and MATK genes present in RNA of microvesicles derived from SHEDs after FGF -6 supplementation. Thus, MVs derived from SHEDs can be used as a biomarker for cellular proliferation. 

### Recommendations

We recommend that in order to properly verify SHEDs as mesenchymal stem cells not dental pulp fibroblast: STRO1, NANOG, SOX family, OCT4 genes needs to be identified along the experimental process, and multipotential differentiation test should be carried out.

Additionally, future research should take into consideration the differentiation potential of these stem cells derived microvesicles and how they compare to the SHEDs differentiation potential before and after growth factor application, utilizing and comparing different isolation protocols for microvesicles, and testing more cargo genes and CDs.

Furthermore, a future protocol should be formulated and tested to utilize microvesicles derived from stem cells as a biomarker for measuring stem cells’ proliferation, via identifying and measuring expression of the genes associated with proliferation, as well as identifying the genes associated with stem cells’ differentiation. That future protocol will be applicable in clinical research that involve stem cells, such as hematopoietic stem cell transplantation, as well as preclinical tissue regeneration experiments such as bone, periodontal, neural regeneration, and regenerative endodontics.

## Data availability

### Underlying data

**Dataset 1. Raw data for the MTT cell viability assay and for reverse transcription-quantitative PCR. DOI:** https://doi.org/10.6084/m9.figshare.11666460.v1 ^30^.
