# Supplementary material for: Fibroblast growth factor-6 enhances CDK2 and MATK expression in microvesicles derived from human stem cells extracted from exfoliated deciduous teeth
Source: F1000Res. 2020 Jun 24;7:622. Originally published 2018 May 22. [Version 6] doi: 10.12688/f1000research.14900.6 (PMC7255775; doi:10.12688/f1000research.14900.6)
Supplement: Peer review history

# Peer review: Reviewer response for version 5

**DOI:** 10.5256/f1000research.26759.r63801

**Published:** 2020-06-11

**Authors:** Thanaphum Osathanon

**Affiliations:** 1 Center of Excellence for Regenerative Dentistry, Faculty of Dentistry, Chulalongkorn University, Bangkok, Thailand

In the revised version, the authors did not respond to all issues raised by the reviewer. Please respond to or rebut each item separately. 

 According to Dominici *et al.* (2006) ^1^, mesenchymal stem cells can be characterized by 1) plastic adherence properties, 2) surface marker expression, and 3) multipotential differentiation ability. This article lacks of mutipotential differentiation test. Hence, the claim of these cells as stem cells isolated from human exfoliated deciduous teeth was not accurate. Indeed, these cells may be dental pulp fibroblast cells. **If authors cannot provide the evidences of MSCs in this article or citation from authors previous publication, these cells can be call dental pulp cells.**

 Authors did not mention which time points that they evaluated for cell viability and proliferation. To determine cell proliferation, at least 2 time points are required and the second time points should be longer than the normal doubling time of these cells.

 Please provide more detail on those reagent used in the study. The catalog number as well as company is crucial information for others to replicate these finding. The main reagents (for example FGF6) were lacking this information.

 In TEM results, it seems that there were a lot of contaminated substance in MV collection. Would this effect the evaluation of gene expression since the RNA from the cells might also contaminated with MVs RNA?

 In PCR results, it seems that there was no statistically significant difference. This might be due to the different baseline expression among donors. Plots demonstrated the individual data could help to visualize the trend between control and treatment in each donors.

 Please indicate n in each experiment. **By this "n" meaning the biological replication of the experiments. Please indicate in each experiment. Authors can place it at the figure legend.**

 Overall, the results seems preliminary and required further improvement to strongly support the conclusion claimed by authors.

Is the work clearly and accurately presented and does it cite the current literature?

No

If applicable, is the statistical analysis and its interpretation appropriate?

Yes

Are all the source data underlying the results available to ensure full reproducibility?

No

Is the study design appropriate and is the work technically sound?

No

Are the conclusions drawn adequately supported by the results?

No

Are sufficient details of methods and analysis provided to allow replication by others?

No

Reviewer Expertise:

Dental stem cells.

I confirm that I have read this submission and believe that I have an appropriate level of expertise to state that I do not consider it to be of an acceptable scientific standard, for reasons outlined above.

## References

[1] : Minimal criteria for defining multipotent mesenchymal stromal cells. The International Society for Cellular Therapy position statement. *Cytotherapy*.2006;8(4) : 10.1080/14653240600855905 315-317 10.1080/14653240600855905 16923606

# Peer review: Reviewer response for version 5

**DOI:** 10.5256/f1000research.26759.r63799

**Published:** 2020-05-22

**Authors:** Abbas Ali Khademi

**Affiliations:** 1 Department of Endodontics, School of Dentistry, Isfahan University of Medical Sciences, Isfahan, Iran

The revised version seems satisfactory.

Is the work clearly and accurately presented and does it cite the current literature?

Yes

If applicable, is the statistical analysis and its interpretation appropriate?

Yes

Are all the source data underlying the results available to ensure full reproducibility?

Yes

Is the study design appropriate and is the work technically sound?

Yes

Are the conclusions drawn adequately supported by the results?

Yes

Are sufficient details of methods and analysis provided to allow replication by others?

Yes

Reviewer Expertise:

Endodontics.

I confirm that I have read this submission and believe that I have an appropriate level of expertise to confirm that it is of an acceptable scientific standard.

# Peer review: Reviewer response for version 4

**DOI:** 10.5256/f1000research.24301.r62082

**Published:** 2020-05-05

**Authors:** Abbas Ali Khademi

**Affiliations:** 1 Department of Endodontics, School of Dentistry, Isfahan University of Medical Sciences, Isfahan, Iran

This study is about evaluation of the effect of FGF-6 on SHEDs and assessment of CDK2 and MATK gene expression, in which the authors reported that CDK2 and MATK genes in SHED-derived can be used as molecular biomarkers for proliferation.

1. Add mean and SD of patients and add female to male ratio or male to female ratio.
2. Please provide approval code or public link.
3. For characterization only flow cytometry is not enough, RT-PCR on genes that show mesenchymal stem cells have been isolated would be interested, e.g. STRO1, NANOG, SOX family, OCT4.
  Or differentiation to osteoblast, chondroblast and adipocytes.
4. Please add a paragraph that show how your results would be helpful to clinical research.
5. Increase the quality of figures.
6. In Figure 3 for CDK2 there are two outliers data.
7. Figure 4 can be removed.

Authors can report the correlation as follows in the results section:

 Expression of CDK2 is positively correlated with cell proliferation in the test group (r=?, P_value:?)

Is the work clearly and accurately presented and does it cite the current literature?

Yes

If applicable, is the statistical analysis and its interpretation appropriate?

Yes

Are all the source data underlying the results available to ensure full reproducibility?

Yes

Is the study design appropriate and is the work technically sound?

Yes

Are the conclusions drawn adequately supported by the results?

Yes

Are sufficient details of methods and analysis provided to allow replication by others?

Yes

Reviewer Expertise:

Endodontics.

I confirm that I have read this submission and believe that I have an appropriate level of expertise to confirm that it is of an acceptable scientific standard, however I have significant reservations, as outlined above.

# Response

**Published:** 2020-05-21

**Authors:** Ahmed Othman

**Affiliations:** Cairo University, Egypt

Dear Dr. Abbas Ali Khademi,

I would like to thank you very much for your review. I truly appreciate your time and effort. We did the following modifications to the article as requested,

The Recommendation section was created to state any future experiment carried out should include a multipotential differentiation test and more genes testing to correctly identify adult stem cells to avoid confusion with dental pulp fibroblasts.

Additionally, a paragraph about potential use in clinical research has been added.

In the correlation section, we added (r) and (P) values.

We added sample size number (n) in the cells viability section.

We rectified the mistake of adding the GAPDH sequence instead of a beta-actin sequence, by adding beta-actin sequence and accession number.

We also recommend in the future to take into consideration male to female ratio and standard deviation during sample collection.

We tried to increase the quality of the figures but unfortunately, we could not, because of the status of equipment during the experiment. In future experiments, we will certainly take that into consideration.

The ethics approval has been provided to F1000Research editors before publishing.

Unfortunately, the funding for the experiment was very limited, so I could not do the multipotential differentiation test or test the presence of STRO1, NANOG, SOX family, OCT4 genes. In the future, we will apply for a much larger fund to conduct the experiments more properly.

My best regards

# Peer review: Reviewer response for version 4

**DOI:** 10.5256/f1000research.24301.r62084

**Published:** 2020-04-14

**Authors:** Thanaphum Osathanon

**Affiliations:** 1 Center of Excellence for Regenerative Dentistry, Faculty of Dentistry, Chulalongkorn University, Bangkok, Thailand

The present article described the effect of FGF6 on RNA contents of microvesicles. In addition, the correlation of detected RNA on cell proliferation was described. There are many issues required to address in order to improve scientific content of this article.

1. According to Dominici *et al.* (2006) ^1^, mesenchymal stem cells can be characterized by 1) plastic adherence properties, 2) surface marker expression, and 3) multipotential differentiation ability. This article lacks of mutipotential differentiation test. Hence, the claim of these cells as stem cells isolated from human exfoliated deciduous teeth was not accurate. Indeed, these cells may be dental pulp fibroblast cells.
2. Authors did not mention which time points that they evaluated for cell viability and proliferation. To determine cell proliferation, at least 2 time points are required and the second time points should be longer than the normal doubling time of these cells.
3. Please provide more detail on those reagent used in the study. The catalog number as well as company is crucial information for others to replicate these finding. The main reagents (for example FGF6) were lacking this information.
4. How many cells were seeded in the tissue culture plates and treated with FGF6 for further cell viability test and MV isolation? Authors mentioned 10 ^3^-10 ^5 ^cells and this is a wide range. Different cell density could reflect the different results obtained.
5. In PCR, authors indicated the use of beta-actin as reference gene but provided GAPDH sequence.
6. In TEM results, it seems that there were a lot of contaminated substance in MV collection. Would this effect the evaluation of gene expression since the RNA from the cells might also contaminated with MVs RNA?
7. In PCR results, it seems that there was no statistically significant difference. This might be due to the different baseline expression among donors. Plots demonstrated the individual data could help to visualize the trend between control and treatment in each donors.
8. What is the r for correlation analysis? Please indicate the statistical number.
9. Please indicate n in each experiment.
10. Overall, the results seems preliminary and required further improvement to strongly support the conclusion claimed by authors.

Is the work clearly and accurately presented and does it cite the current literature?

No

If applicable, is the statistical analysis and its interpretation appropriate?

Yes

Are all the source data underlying the results available to ensure full reproducibility?

No

Is the study design appropriate and is the work technically sound?

No

Are the conclusions drawn adequately supported by the results?

No

Are sufficient details of methods and analysis provided to allow replication by others?

No

Reviewer Expertise:

Dental stem cells.

I confirm that I have read this submission and believe that I have an appropriate level of expertise to state that I do not consider it to be of an acceptable scientific standard, for reasons outlined above.

## References

[1] : Minimal criteria for defining multipotent mesenchymal stromal cells. The International Society for Cellular Therapy position statement. *Cytotherapy*.2006;8(4) : 10.1080/14653240600855905 315-7 10.1080/14653240600855905 16923606

# Response

**Published:** 2020-05-21

**Authors:** Ahmed Othman

**Affiliations:** Cairo University, Egypt

Dear Dr. Thanaphum Osathanon,

I would like to thank you very much for your review. I truly appreciate your time and effort. We did the following modifications to the article as requested,

The Recommendation section was created to state any future experiment carried out should include a multipotential differentiation test and more genes testing to correctly identify adult stem cells to avoid confusion with dental pulp fibroblasts.

Additionally, a paragraph about potential use in clinical research has been added.

In the correlation section, we added (r) and (P) values.

We added sample size number (n) in the cells viability section.

We rectified the mistake of adding the GAPDH sequence instead of a beta-actin sequence, by adding beta-actin sequence and accession number.

We also recommend in the future to take into consideration male to female ratio and standard deviation during sample collection.

We tried to increase the quality of the figures but unfortunately, we could not, because of the status of equipment during the experiment. In future experiments, we will certainly take that into consideration.

The ethics approval has been provided to F1000Research editors before publishing.

Unfortunately, the funding for the experiment was very limited, so I could not do the multipotential differentiation test or test the presence of STRO1, NANOG, SOX family, OCT4 genes. In the future, we will apply for a much larger fund to conduct the experiments more properly.

We contacted the lab to query about FGF-6, but unfortunately, they are unreachable because of lockdown in the university. As soon as I know the manufacturer and catalog number, I will update the article.

My best regards

# Peer review: Reviewer response for version 4

**DOI:** 10.5256/f1000research.24301.r59229

**Published:** 2020-02-04

**Authors:** Asma Abdullah Nurul

**Affiliations:** 1 School of Health Sciences, University of Science Malaysia, Kubang Kerian, Malaysia

The authors have responded to the comments given in the previous review.

Is the work clearly and accurately presented and does it cite the current literature?

Yes

If applicable, is the statistical analysis and its interpretation appropriate?

Partly

Are all the source data underlying the results available to ensure full reproducibility?

Partly

Is the study design appropriate and is the work technically sound?

Yes

Are the conclusions drawn adequately supported by the results?

Partly

Are sufficient details of methods and analysis provided to allow replication by others?

Yes

Reviewer Expertise:

Stem cell, regenerative medicine, molecular immunology

I confirm that I have read this submission and believe that I have an appropriate level of expertise to confirm that it is of an acceptable scientific standard.

# Peer review: Reviewer response for version 3

**DOI:** 10.5256/f1000research.21423.r52495

**Published:** 2019-09-18

**Authors:** Asma Abdullah Nurul

**Affiliations:** 1 School of Health Sciences, University of Science Malaysia, Kubang Kerian, Malaysia

- In the revision 3, the authors have amended some part of the manuscript, but ignored some of the reviewer's comments.
- The conclusion in the abstract is not corresponded to the main aim of the study.
- Why the authors did not include mRNA expression of CDK2 and MATK from SHED or treated SHED, instead of SHED-derived MVs alone.
- Discussion sounds more like rationale of study, not discussing about the main findings and relate with previous works.
- The current write up is not so impressive, so I recommend re-writing of the manuscript.

Is the work clearly and accurately presented and does it cite the current literature?

Yes

If applicable, is the statistical analysis and its interpretation appropriate?

Partly

Are all the source data underlying the results available to ensure full reproducibility?

Partly

Is the study design appropriate and is the work technically sound?

Yes

Are the conclusions drawn adequately supported by the results?

Partly

Are sufficient details of methods and analysis provided to allow replication by others?

Yes

Reviewer Expertise:

Stem cell, regenerative medicine, molecular immunology

I confirm that I have read this submission and believe that I have an appropriate level of expertise to state that I do not consider it to be of an acceptable scientific standard, for reasons outlined above.

# Peer review: Reviewer response for version 2

**DOI:** 10.5256/f1000research.16430.r35756

**Published:** 2018-07-16

**Authors:** Asma Abdullah Nurul

**Affiliations:** 1 School of Health Sciences, University of Science Malaysia, Kubang Kerian, Malaysia

The authors have presented a  work on the effects of fibroblast growth factor-6 on the expression of CDK2 and MATK in microvesicles derived from stem cells from exfoliated deciduous teeth (SHED). In the study, the authors have shown that treatment with FBF-6 enhances the proliferative activity as well as the expressions of CDK2 and MATK in MV derived from SHED. This study is somewhat preliminary and the manuscript requires modifications to further improve it.

1. There is inconsistency in the usage of terminology for example MATK was misspelled with MAKT many times (also in the title).
2. Figure 2 should include population gating and isotype control.
3. The authors should provide r value of the correlation coefficients of the CDK2 and MATK expressions to cell proliferative activity as shown in Figure 4. 
4. Conclusion need to be rephrased.
5. The language use in the manuscript is unsuitable for publication. I would like to suggest submitting the manuscript for professional English editing.

Is the work clearly and accurately presented and does it cite the current literature?

Yes

If applicable, is the statistical analysis and its interpretation appropriate?

Partly

Are all the source data underlying the results available to ensure full reproducibility?

Partly

Is the study design appropriate and is the work technically sound?

Yes

Are the conclusions drawn adequately supported by the results?

Partly

Are sufficient details of methods and analysis provided to allow replication by others?

Yes

Reviewer Expertise:

stem cell, regenerative medicine, molecular immunology

I confirm that I have read this submission and believe that I have an appropriate level of expertise to confirm that it is of an acceptable scientific standard, however I have significant reservations, as outlined above.
